# Type I interferonopathies with novel compound heterozygous *TREX1* mutations in two siblings with different symptoms responded to tofacitinib

**DOI:** 10.1186/s12969-020-00490-1

**Published:** 2021-01-06

**Authors:** Shiyu Zhang, Jiaxing Song, Yuyan Yang, Huilei Miao, Lu Yang, Yuehua Liu, Xue Zhang, Yaping Liu, Tao Wang

**Affiliations:** 1 Department of Dermatology, Peking Union Medical College Hospital, Chinese Academy of Medical Sciences and Peking Union Medical College, Beijing, 100730 China; 2 Department of Medical Genetics and National Laboratory of Medical Molecular Biology, Institute of Basic Medical Sciences, Chinese Academy of Medical Sciences and Peking Union Medical College, Beijing, 100005 China; 3 Peking Union Medical College and Chinese Academy of Medical Sciences, Beijing, China

**Keywords:** Aicardi-Goutières syndrome, Compound heterozygote, Familial chilblain lupus, Interferonopathy, Tofacitinib, TREX1

## Abstract

**Background:**

Type I interferonopathies are a group of rare autoimmune diseases characterised by excessive activation of type I interferon that leads to disturbances in immune function. Three prime repair exonuclease 1 (TREX1) is an important exonuclease and plays an important role in DNA damage repair. *TREX1* mutations are associated with many type I interferonopathies. Studies have been published on the effectiveness of tofacitinib in the treatment of type I interferonopathies. The aim of this study is to identify the pathogenic variation in a Chinese family with type I interferonopathies and to observe the therapeutic effects of tofacitinib.

**Methods:**

A Chinese family with two members with type I interferonopathies was investigated. Whole exome sequencing and Sanger sequencing were applied for mutation screening using peripheral blood DNA of the patient and her family members. Sequencing results were analysed using bioinformatics software tools including VarCards and PolyPhen-2. Close clinical follow-up and observation were used to record changes in the disease before and after treatment with tofacitinib.

**Results:**

Compound heterozygous variants of *TREX1* were observed in the patient’s genome. One was a missense variant (NM_016381; c.C227T; p.Ala76Val) from the patient’s father, and the other was a frameshift variant (NM_016381; c.458dupA; p.Gln153Glnfs*3) from the patient’s mother. One of the proband’s elder brothers with similar skin lesions also carried these two variants. This brother of the proband had more serious cutaneous involvement with the comorbidity of cerebral palsy. These *TREX1* variants have not been reported in previous studies and are predicted to be highly pathogenic. The proband was given tofacitinib that led to a marked improvement.

**Conclusions:**

We identified two novel complex heterozygous variants in the *TREX1* gene, which may underlie the molecular pathogenesis of the type I interferonopathies observed in members of this family. Tofacitinib could be an alternative treatment for this disease.

## Background

Type I interferon (IFN) plays an important role in the immune defence against viral infections [1]. Excessive activation of type I IFN can lead to disturbances in immune function. Type I interferonopathies are autoinflammatory and autoimmune disorders [1, 2] characterised by the upregulation of type I IFN. Notwithstanding type I IFN upregulation, each disease in the category of type I interferonopathies has its own clinical manifestations. The main types of diseases and their causative genes are shown in Table 1 [1, 3].

**Table 1:** The main types of type I interferonopathies and their causative genes

Disease	Gene
Aicardi-Goutières syndrome (AGS)	*TREX1, RNASEH2A, RNASEH2B,* *RNASEH2C, SAMHD1, ADAR1, IFIH1*
Retinal vasculopathy with cerebral leukodystrophy	*TREX1*
Familial chilblain lupus (FCL)	*TREX1, SAMHD1, STING*
Systemic lupus erythematosus	*TREX1, RNASEH2A-C, ACP5, DNASE1, DNASE1L3, C1QA-C, C4*
STING-associated vasculopathy, infantile-onset	*STING*
Singleton-Merten syndrome	*IFIH1, RIGI*
Spondyloenchrondrodysplasia	*ACP5*
ISG15 deficiency	*ISG15*
USP18 deficiency	*USP18*
Chronic atypical neutrophilic dermatosis with lipodystrophy and elevated temperature	*PSMB8, PSMB4, PSMA3, PSMB9, POMP*
X-linked reticulate pigmentary disorder	*POLA1*
Panarteritis nodosa, childhood-onset	*CECR1*

Abbreviations: *AGS* Aicardi-Goutières syndrome; *FCL* familial chilblain lupus

Three prime repair exonuclease 1 (TREX1) plays an important role in DNA damage repair [1, 4]. A growing number of studies have shown that *TREX1* mutations are associated with multiple type I interferonopathies [1, 5]. Aicardi-Goutières syndrome (AGS) is a subgroup of type I interferonopathies [1, 3, 6]. Familial chilblain lupus (FCL) is a subtype of AGS characterised by papular skin lesions, which occur as a result of the cold [1]. FCL affects the tip of the finger or toe and the folds of the nail, and there is usually no associated neurological condition [1, 7]. The pathogenic genes of FCL include *TREX1*, *SAMHD1 and STING*. Tofacitinib, a Janus kinase (JAK) inhibitor, has been described to improve type I interferonopathies by suppressing the overactivated JAK / signal transducing activator of transcription (STAT) pathway.

In this article, we describe the clinical, pathological and genetic characteristics of two cases with complex heterozygous variants in the *TREX1* gene. These two variants have not been reported in previous studies and have not been reported as a polymorphic change in public databases; the frequency of the variants in the population is extremely low. Tofacitinib treatment led to an improvement in the patient.

## Materials and methods

### Participants

**Fig. 1:**
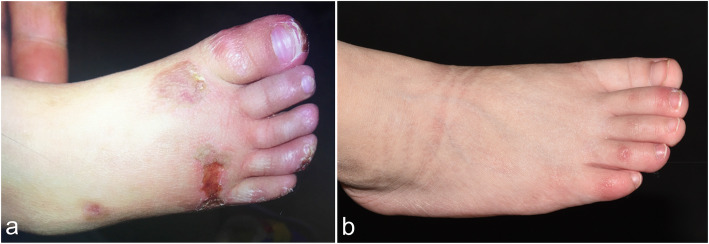
Clinical manifestations on the foot of the proband. **a** Chilblain lesions on the toes and the right dorsal feet. **b** After treatment, the skin lesions were controlled

**Fig. 2:**
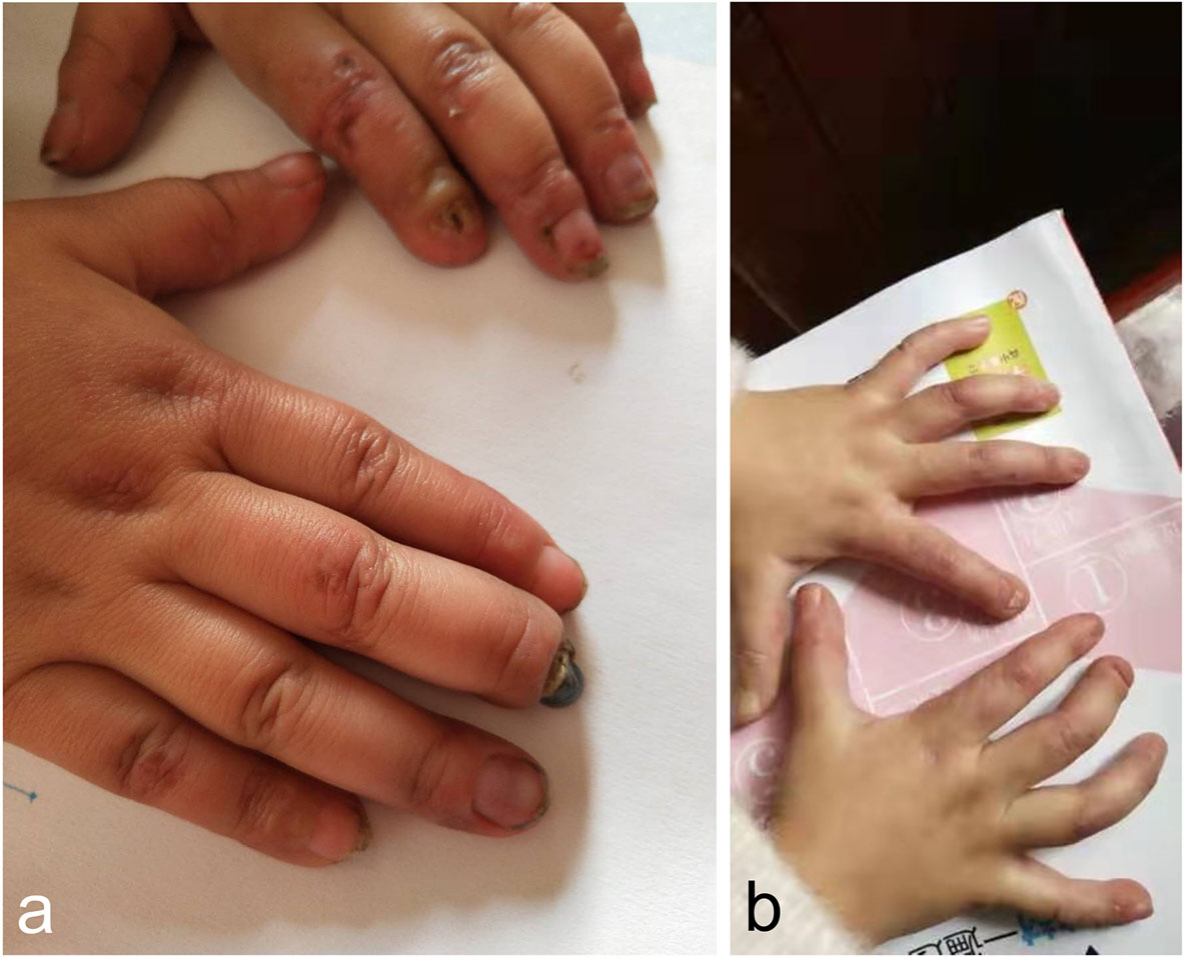
Clinical manifestations on the hands of the proband. **a** Chilblain lesions on the fingers. **b** After treatment, the skin lesions were controlled

**Fig. 3:**
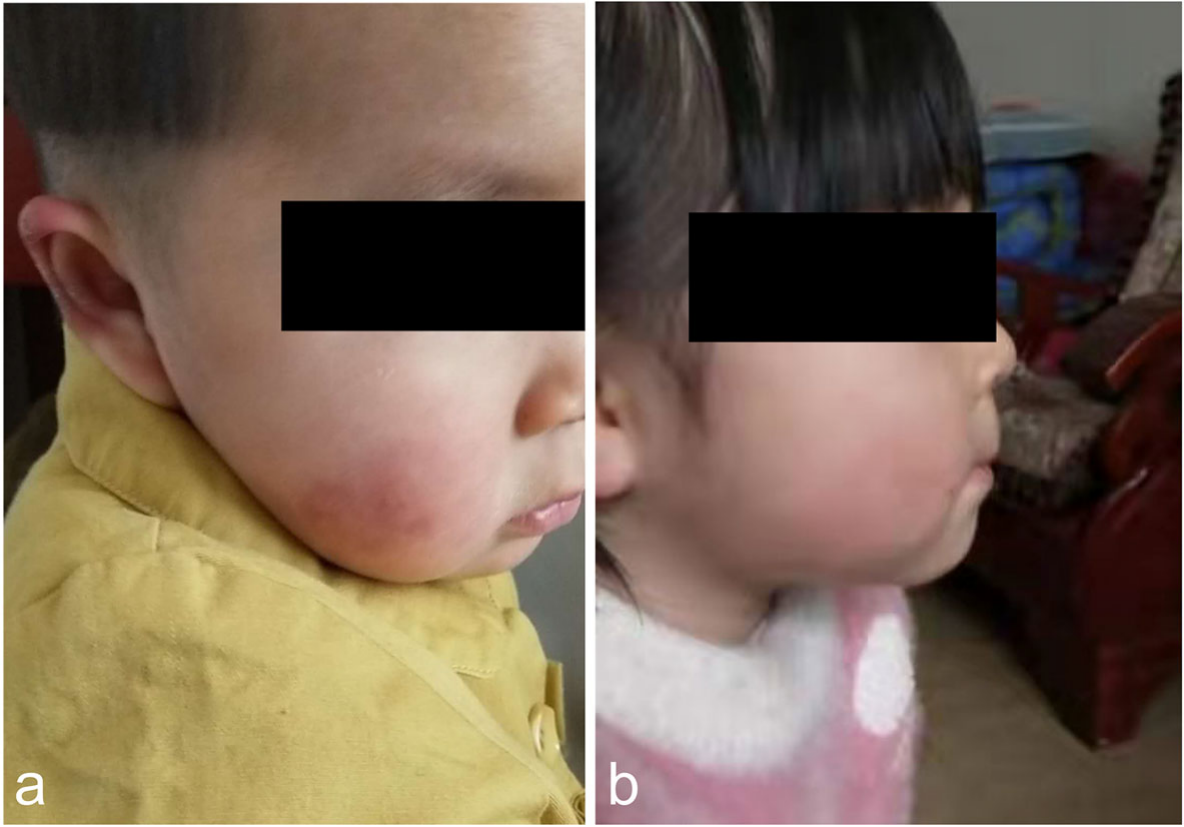
Clinical manifestations on the face of the proband. **a** Chilblain lesions on the face. **b** After treatment, the skin lesions were controlled

A 2-year-old female was admitted to our outpatient clinic in the Department of Dermatology due to painful bluish-red papular lesions of the skin in acral locations. The erythematous infiltrates first occurred when she was 8 months old, affecting mainly the interphalangeal joints of her fingers, toes and ears. Ulcerations were commonly noted in distal portions of her toes and fingers, causing nail changes. Scars were left without blisters or necrotic areas (Figs. 1a and 2a). Erythematous to violaceous indurated infiltrative plaques appeared on the face (Fig. 3a).

**Fig. 4:**
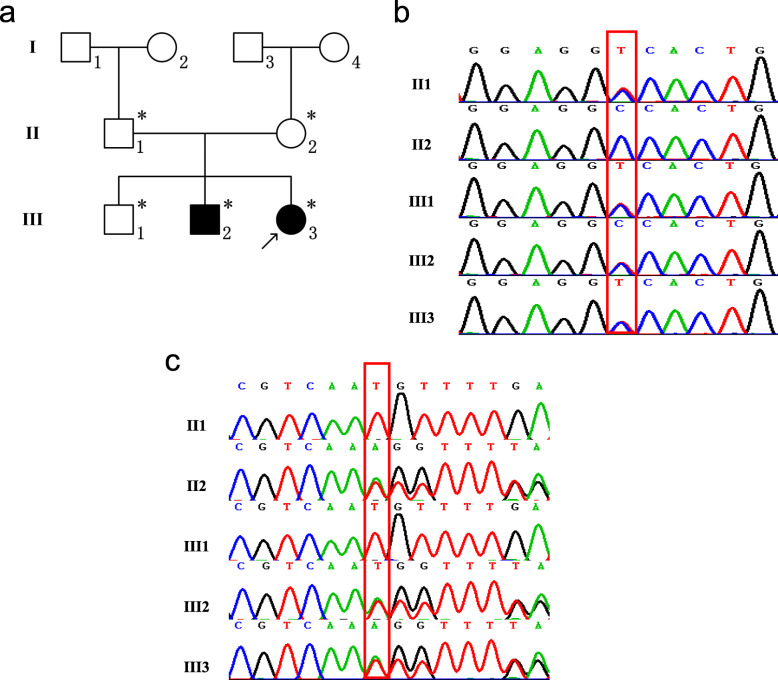
Pedigree of the family and Sanger sequencing results and identified mutations. **a** Pedigree of the family with FCL. The arrow indicates the proband, and the asterisks indicate family members who had genetic testing in this study. **b, c** Sanger sequencing results and identified mutations in pedigree. (**b**: TREX1 NM_016381; c.C227T; p.Ala76Val. **c**: TREX1 NM_016381; c.458dupA; p.Gln153fs3)

She is the 3rd child of a healthy non-consanguineous couple with no family history of haemophilia or skin diseases (Fig. 4a). Her eldest brother was in good health, while her elder brother presented similar skin lesions, albeit much more severe, at the same age, with necrotic destruction leading to the falling off of his fingers. In addition to the skin disorder, he also had cerebral-palsy-like abnormality in motor development and posture.

**Fig. 5:**
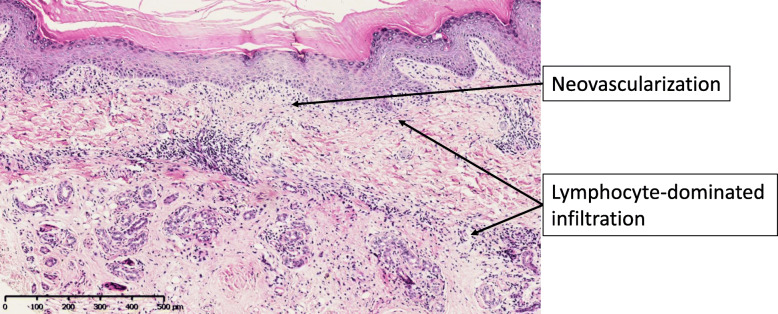
Histopathological findings of a skin lesion of the proband. Histopathological examination showed significant hyperkeratosis and acanthosis. There were lymphocyte-dominated inflammatory infiltration at the dermal junction and around the dermal vessels and adnexa, and neovascularisation in the dermal papilla (H&E, × 100)

A skin biopsy taken from the borders of an erythematous lesion upon her right foot showed significant hyperkeratosis and acanthosis. There was lymphocyte-dominated inflammatory infiltration at the dermal junction and around the dermal vessels and adnexa, and neovascularisation in the dermal papilla (Fig. 5).

Participants and patients in this study have given written informed consent to be in the study and to allow the publication of their case details. The ethics review board of the institution approved the study.

### DNA sample preparation

Genomic DNA was extracted from peripheral blood using the QIAamp DNA Blood Mini Kit (QIAGEN, Hilden, Germany), according to the standard protocol, and quantified using a NanoDROP 2000 spectrophotometer (Thermo Scientific; Waltham, MA, USA).

### Mutational analysis of the *TREX1* gene

Whole exome sequencing (WES) was conducted in the proband by the Novogene company on the Illumina NovaSeq platform, with an average sequencing depth of 100X. Raw sequence results were aligned to the human reference genome (GRCh37/hg19) and annotated to obtain the candidate variants. Then the candidate variants were validated using Sanger sequencing to confirm the WES results. Primers were designed using Primer3web, version 4.1.0 for the suspected disease-causing genes. Variants were interpreted using VarCards (http://varcards.biols.ac.cn/) and PolyPhen2 (http://genetics.bwh.harvard.edu/pph2/). And we also used SWISS-MODEL (https://swissmodel.expasy.org/) to predict the effect of the two mutations on protein structure.

#### Measuring the expression levels of IFN-stimulated genes in the patient

Real-time quantitative PCR was performed to measure the expression levels of five IFN-stimulated genes (ISGs) (*IFI27, IFI44L, IFIT1, ISG15, RSAD2*) in the patient after treatment. The results were compared with the expression levels of these genes in normal controls.

## Results

**Fig. 6:**
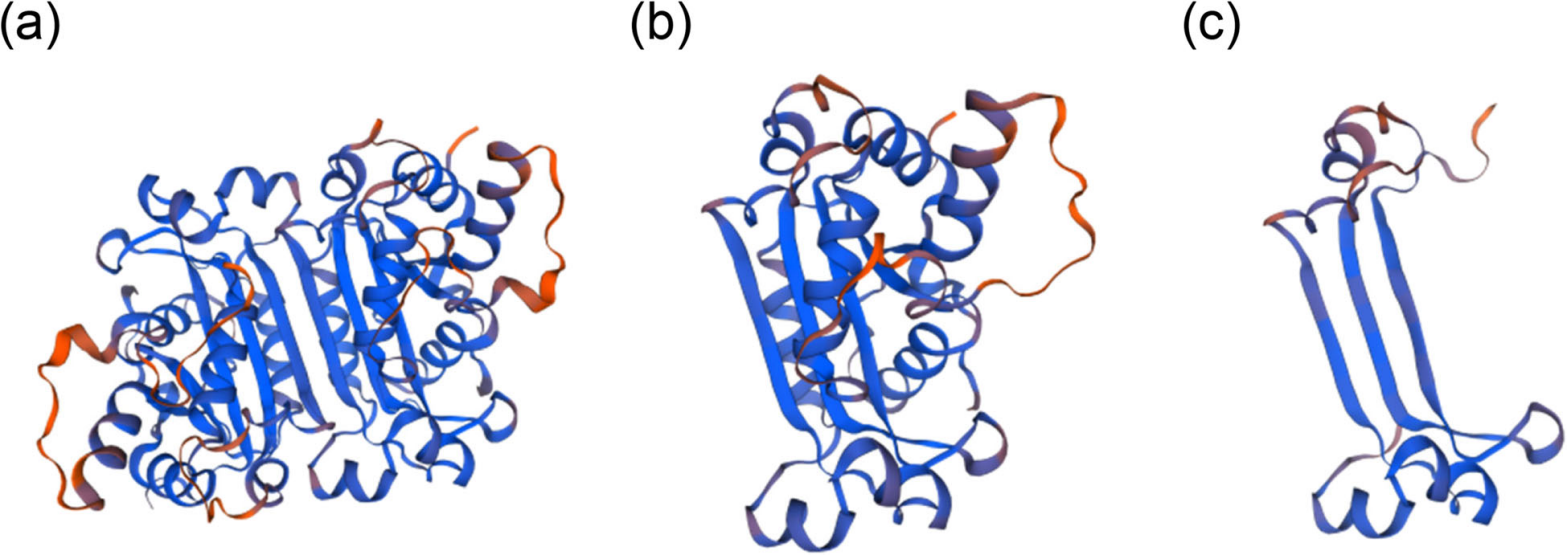
Effects of the mutations in *TREX1* in the patient were simulated by means of SWISS-MODEL. **a** Predicted normal TREX1 protein structure. **b** Predicted TREX1 protein structure with a specific missense mutation. **c** Predicted TREX1 protein structure with a specific frameshift mutation

**Fig. 7:**
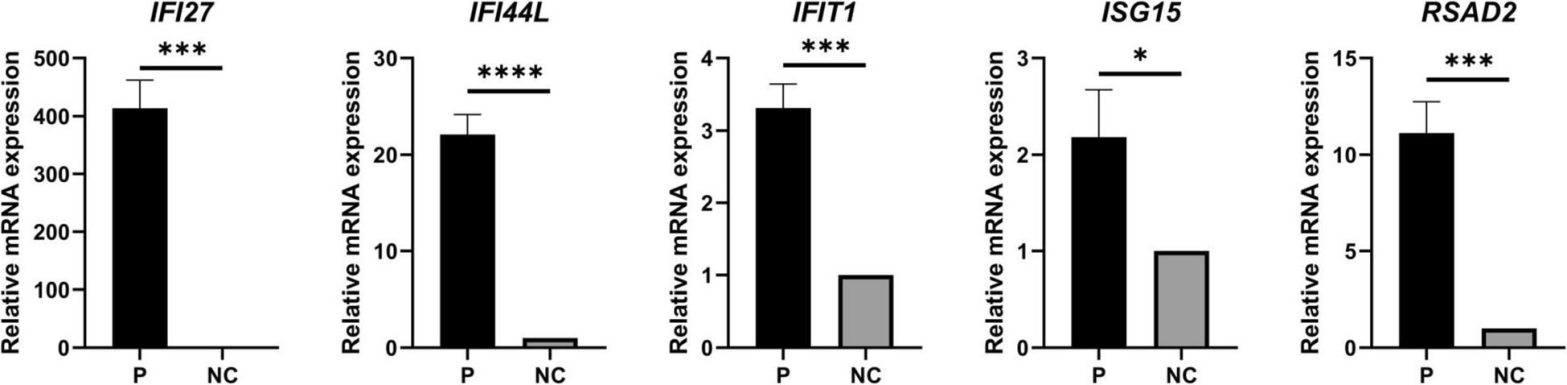
Targeted IFN-stimulated gene RNA expression in total blood in patient compared with normal control. The ratio of the gene of interest to *GAPDH* in the normal control was set to 1. Data shown were mean ± SD of 3 three experiments, and each experiment was performed in duplicate. P: Patient, NC: normal control

By analysing the WES results, we found two variants of the *TREX1* gene. One was a missense variant (NM_016381; c.C227T; p.Ala76Vla), and the other was a frameshift variant (NM_016381; c.458dupA; p.Gln153Glnfs*3). The two variants were verified using Sanger sequencing (Fig. 4b, c). To verify whether the two variants were de novo or not, the patients’ parents were tested using Sanger sequencing. As shown in Fig. 4b, the father of the proband was a heterozygote of the variant c.C227T, and the mother was a heterozygote of the variant c.458dupA (Fig. 4c). Both mutations were novel. The proband’s affected brother carried the same variants as the proband, so the inheritance was in accordance with an autosomal recessive pattern. The damaging score of the missense mutation was 0.83 (damaging score of loss-of-function variant is deemed to be 1) by using VarCards and this mutation was predicted to be probably damaging with a score of 1.000 by using PolyPhen2. According to the SWISS-MODEL prediction, the overall structure of the protein with either missense mutation or frameshift mutation of *TREX1* had undergone tremendous changes (Fig. 6), which indicated that these two mutations would affect the structure and function of the protein. Real-time quantitative PCR revealed significantly elevated expression levels of five ISGs (*IFI27, IFI44L, IFIT1, ISG15, RSAD2*) in the patient than in the normal controls (Fig. 7). Accordingly, the patient and her affected brother were diagnosed with FCL.

After the diagnosis was made, the proband was given tofacitinib 2.5 mg bid, which led to marked improvement (Figs. 1b, 2b and 3b). During the following 2 years, the skin lesions and fever could be controlled with tofacitinib alone during the summer and autumn, but got worse during the winter with arthralgia, which could be controlled with tofacitinib 2.5 mg bid and prednisone 5 mg qd. No mild or severe adverse events were observed. She currently lives a normal kindergarten life.

## Discussion

In this study, we found two unreported mutations in *TREX1* in a Chinese family. One mutation was a missense mutation (NM_016381; c.C227T; p.Ala76Val), and the other was a frameshift mutation (NM_016381; c.458dupA; p. Gln153Glnfs*3). Previously, researchers had reported on the mechanism of *TREX1* causing type I interferonopathies [8]. Diseases associated with *TREX1* are shown in Table 2.

**Table 2:** Diseases associated with *TREX1*

Disease	Inheritance	Clinical manifestation	Treatment
Aicardi-Goutières syndrome 1 (AGS1)	AD/AR	Progressive encephalopathy, developmental delay, deformity, foot bun, frostbite, purpura	NSAIDs, JAK inhibitor, reverse-transcriptase inhibitors
Familial chilblain lupus (FCL)	AD/AR	Painful blue-red papules or nodules (finger, toes, nose, cheeks, ears), skin ulcers after exposure to cold and wet	Hydroxychloroquine, corticosteroids, cyclophosphamide, mycophenolate mofetil, JAK inhibitor
Vasculopathy, retinal, with cerebral leukodystrophy	AD	Retinal vasculopathy, Raynaud phenomenon, CNS degradation	Corticosteroids, NSAIDs, clopidogrel, heparin, levetiracetam
Systemic lupus erythematosus, susceptibility to	AD	Fatigue, fever, arthritis, mucocutaneous manifastations, renal, hematologic, CNS involvement and other systemic involvement	Hydroxychloroquine, NSAIDs, corticosteroids, methotrexate

Abbreviations: *AD* autosomal dominant; *AGS* Aicardi-Goutières syndrome; *AR* autosomal recessive; *FCL* familial chilblain lupus; *JAK* Janus-kinases; *NSAIDs* nonsteroidal anti-inflammatory drugs

The correlation of gene mutations and clinical manifestations of the patients was consistent with the diagnosis of FCL, a subtype of AGS and one of the type I interferonopathies. The proband and her brother carried the same mutations, but her brother had neurological symptoms in addition to the symptoms on the skin. This was also in consistence with the fact that a deficient activity of *TREX1* could affect both the nervous system and the skin, which led to corresponding manifestations in AGS patients.

The proband was given tofacitinib to treat her FCL symptoms and showed improvement. This was consistent with recent studies revealing that JAK or reverse transcriptase inhibitors could benefit patients with type I interferonopathies [9–13]. König et al. reported a family with five members affected by FCL. Two of the members were treated with tofacitinib 5 mg twice daily for 17 days. On the 14th day of treatment, the IFN signature was significantly suppressed, and the pain and discomfort of the fingers were relieved [11]. The mechanism of the therapeutic effects of tofacitinib in interferonopathies might be that the tofacitinib inhibits the abnormally activated IFN-α/β receptor, the JAK/STAT pathway, and the subsequent autoinflammation [11, 14]. In the CD4 T cells and CD19 B cells from 2 patients with mutation of stimulator of interferon genes (STING) treated with tofacitinib, the phosphorylation of *STAT1*, *STAT2* and *STAT3* was blocked [14]. Tofacitinib opened up a new choice of treatment for one of the affected members of the family in this study.

The limitation of this study is that we did not measure the changes in type I IFN in the patient before the treatment by JAK inhibitor. We sampled the peripheral blood of the patient at his first visit but didn’t extract the RNA immediately so that RNA was all gone after it had been stored for too long. Due to the extremely low concentration of type I interferon in blood, it can’t be detected directly by using conventional ELISA. Currently, the expression levels of five IFN-stimulated genes (ISGs) (*IFI27, IFI44L, IFIT1, ISG15, RSAD2*) are generally used to assess the activity of type I interferon signaling pathway [15–17] by real-time quantitative PCR. Here, to provide the supportive evidence for the pathogenicity of the novel compound heterozygous *TREX1* mutations, we measured the differences of five ISGs between the patient after treatment and normal controls (Fig. 7). The expression level of the five ISGs of the patient was higher than that of the normal control. Studies have confirmed that the expression of ISGs in patients with type I interferonopathies would have a significant decline before and after treatment by JAK inhibitor [18]. In our study, as shown in Fig. 7, although we only measured the changes of five ISGs in this patient after treatment, we reasonably speculated that the expression level of ISGs in this patient before treatment was also elevated and had be higher than that after treatment. Therefore, this result can be an indirect line of evidence to support the genetic diagnosis of this patient.

This study expanded the mutation spectrum of the *TREX1* gene and provided an accurate genetic diagnosis for the affected members of the family. The accuracy of the diagnosis has greatly helped the dermatologists to make decisions on the patient’s follow-up treatment, especially for the skin symptoms. In clinical practice, the same or similar symptoms can occur in a variety of skin diseases, which greatly adds to the difficulty in making the correct skin disease diagnosis. The emergence of WES has greatly helped physicians in the diagnosis and treatment of skin diseases. WES has proved to be a valuable tool in the diagnosis and research of hereditary skin diseases such as interferonopathies and is worthwhile performing.

## Conclusions

In summary, we identified two novel complex heterozygous variants in the *TREX1* gene, which may participate in the pathogenesis of the type I interferonopathies observed in patients in this family. Tofacitinib could be an alternative treatment for this disease.

## Data Availability

The datasets used and/or analysed during the current study are available from the corresponding author on reasonable request.

## Abbreviations

AGS: Aicardi-Goutières syndrome
FCL: Familial chilblain lupus
IFN: Interferon
JAK: Janus-kinases
STAT: Signal transducing activator of transcription
STING: Stimulator of interferon genes
TREX1: Three prime repair exonuclease 1
WES: Whole exome sequencing
